# Supplementary material for: Transient effect of melatonin treatment after neonatal hypoxic-ischemic brain injury in rats
Source: PLoS One. 2019 Dec 20;14(12):e0225788. doi: 10.1371/journal.pone.0225788 (PMC6924669; doi:10.1371/journal.pone.0225788)
Supplement: S1 Table — (DOCX) [file pone.0225788.s001.docx]

**Supplementary Table 1. Results for clusters of animals with mild and severe injuries.**

|  | **Days after HI** | **Mild** | |  | **Severe** | |  |
| --- | --- | --- | --- | --- | --- | --- | --- |
|  |  | **mean** | **S.E.M.** |  | **mean** | **S.E.M.** | **P-value** |
| **T2w images** |  |  |  |  |  |  |  |
| Intact ratio | 1 | 1.01 | 0.01 |  | 0.63 | 0.03 | **<0.001** |
|  | 7 | 0.72 | 0.05 |  | 0.33 | 0.02 | **<0.001** |
|  | 20 | 0.73 | 0.05 |  | 0.31 | 0.01 | **<0.001** |
|  | 43 | 0.73 | 0.05 |  | 0.30 | 0.01 | **<0.001** |
| Hypointense ipsilateral volume (uL) | 1 | 0.0 | 0.0 |  | 0.0 | 0.0 | NA |
|  | 7 | 12.8 | 3.5 |  | 20.6 | 1.2 | 0.077 |
|  | 20 | 6.8 | 2.3 |  | 11.7 | 1.1 | 0.085 |
|  | 43 | 7.7 | 3.2 |  | 7.8 | 1.0 | 0.287 |
| Hyperintense ispilateral volume (uL) | 1 | 3.7 | 1.7 |  | 118.1 | 8.9 | **<0.001** |
|  | 7 | 15.8 | 7.2 |  | 147.4 | 8.1 | **<0.001** |
|  | 20 | 4.4 | 2.5 |  | 178.4 | 17.4 | **<0.001** |
|  | 43 | 9.0 | 5.1 |  | 271.0 | 25.2 | **<0.001** |
| Normal ipsilateral volume (uL) | 1 | 311.1 | 10.1 |  | 177.7 | 12.1 | **<0.001** |
|  | 7 | 293.2 | 20.5 |  | 128.4 | 7.1 | **<0.001** |
|  | 20 | 358.9 | 25.7 |  | 147.7 | 7.3 | **<0.001** |
|  | 43 | 401.5 | 31.2 |  | 162.3 | 8.7 | **<0.001** |
| **DTI** |  |  |  |  |  |  |  |
| FA | 7 | 0.509 | 0.017 |  | 0.436 | 0.012 | **<0.01** |
|  | 20 | 0.604 | 0.022 |  | 0.461 | 0.013 | **<0.001** |
|  | 43 | 0.501 | 0.024 |  | 0.422 | 0.011 | **<0.01** |
| MD (um^2/s) | 7 | 1065 | 27.1 |  | 1057 | 20.9 | 0.640 |
|  | 20 | 1071 | 24.8 |  | 1155 | 26.9 | 0.114 |
|  | 43 | 1221 | 40.6 |  | 1249 | 24.3 | 0.390 |
| RD (mm^2/s) | 7 | 730 | 30.7 |  | 794 | 24.7 | 0.121 |
|  | 20 | 646 | 29.1 |  | 850 | 31.1 | **<0.001** |
|  | 43 | 849 | 53.2 |  | 945 | 26.8 | 0.090 |
| L1 (mm^2/s) | 7 | 1735 | 30.7 |  | 1583 | 17.9 | **<0.001** |
|  | 20 | 1923 | 62.1 |  | 1765 | 26.0 | **<0.05** |
|  | 43 | 1964 | 26.4 |  | 1855 | 27.7 | **<0.01** |
| **Histology** |  |  |  |  |  |  |  |
| Histology score | 43 | 10.0 | 0.6 |  | 3.1 | 0.3 | **<0.001** |
|  |  |  |  |  |  |  |  |
| **Functional tests** |  |  |  |  |  |  |  |
| Cylinder rearing |  |  |  |  |  |  |  |
| RFP | 15 | 0.58 | 0.11 |  | 0.77 | 0.05 | 0.151 |
|  | 36 | 0.60 | 0.02 |  | 0.71 | 0.03 | 0.147 |
| Novel object recognition | |  |  |  |  |  |  |
| D1-1h (s) | 15 | 6.7 | 1.0 |  | 0.2 | 3.7 | 0.476 |
| D1-24h (s) | 15 | -2.8 | 2.2 |  | -1.8 | 1.0 | 0.609 |
| D1-1h (s) | 36 | 9.2 | 3.4 |  | 5.9 | 2.1 | 0.424 |
| D1-24h (s) | 36 | 5.2 | 3.4 |  | 5.7 | 1.9 | 0.961 |
| ET-1h (s) | 15 | 11.5 | 1.5 |  | 3.6 | 1.1 | **<0.05** |
| ET-24h (s) | 15 | 6.2 | 1.8 |  | 2.7 | 0.6 | 0.121 |
| ET-1h (s) | 36 | 28.8 | 5.5 |  | 22.6 | 2.5 | 0.262 |
| ET-24h (s) | 36 | 16.4 | 5.7 |  | 16.5 | 2.4 | 0.961 |
