# Supplementary material for: Transient effect of melatonin treatment after neonatal hypoxic-ischemic brain injury in rats
Source: PLoS One. 2019 Dec 20;14(12):e0225788. doi: 10.1371/journal.pone.0225788 (PMC6924669; doi:10.1371/journal.pone.0225788)
Supplement: S2 Table — (DOCX) [file pone.0225788.s002.docx]

**Supplementary Table 2. Results for male and female animals.**

|  | **Days after HI** | **Male** | |  | **Female** | |  |
| --- | --- | --- | --- | --- | --- | --- | --- |
|  |  | **mean** | **S.E.M.** |  | **mean** | **S.E.M.** | **P-value** |
| **T2w images** |  |  |  |  |  |  |  |
| Intact ratio | 1 | 0.75 | 0.04 |  | 0.74 | 0.07 | 0.938 |
|  | 7 | 0.41 | 0.04 |  | 0.49 | 0.06 | 0.324 |
|  | 20 | 0.40 | 0.04 |  | 0.47 | 0.07 | 0.366 |
|  | 43 | 0.40 | 0.05 |  | 0.46 | 0.07 | 0.427 |
| Hypointense ipsilateral volume (uL) | 1 | 0.0 | 0.0 |  | 0.0 | 0.0 | N.A. |
|  | 7 | 19.4 | 2.0 |  | 17.1 | 2.2 | 0.459 |
|  | 20 | 11.4 | 1.6 |  | 9.2 | 1.5 | 0.331 |
|  | 43 | 8.6 | 1.7 |  | 7.0 | 1.6 | 0.516 |
| Hyperintense ispilateral volume (uL) | 1 | 84.9 | 14.1 |  | 82.7 | 18.8 | 0.927 |
|  | 7 | 119.6 | 17.2 |  | 96.3 | 18.8 | 0.369 |
|  | 20 | 136.9 | 26.4 |  | 115.4 | 28.3 | 0.584 |
|  | 43 | 207.3 | 37.9 |  | 177.5 | 43.3 | 0.608 |
| Normal ipsilateral volume (uL) | 1 | 216.9 | 16.4 |  | 218.6 | 24.3 | 0.952 |
|  | 7 | 164.8 | 18.1 |  | 190.8 | 26.6 | 0.426 |
|  | 20 | 194.2 | 21.5 |  | 227.9 | 34.2 | 0.412 |
|  | 43 | 218.7 | 26.7 |  | 249.4 | 38.2 | 0.516 |
| **DTI** |  |  |  |  |  |  |  |
| FA | 7 | 0.465 | 0.016 |  | 0.454 | 0.016 | 0.635 |
|  | 20 | 0.494 | 0.017 |  | 0.507 | 0.028 | 0.692 |
|  | 43 | 0.450 | 0.015 |  | 0.441 | 0.019 | 0.722 |
| MD (um^2/s) | 7 | 1038 | 24.2 |  | 1082 | 19.5 | 0.183 |
|  | 20 | 1130 | 29.2 |  | 1133 | 31.9 | 0.951 |
|  | 43 | 1247 | 31.0 |  | 1233 | 28.3 | 0.752 |
| RD (mm^2/s) | 7 | 754 | 30.4 |  | 792 | 23.9 | 0.351 |
|  | 20 | 797 | 35.5 |  | 790 | 47.0 | 0.917 |
|  | 43 | 917 | 38.2 |  | 916 | 35.0 | 0.986 |
| L1 (mm^2/s) | 7 | 1604 | 21.3 |  | 1660 | 32.7 | 0.177 |
|  | 20 | 1798 | 33.9 |  | 1819 | 45.3 | 0.725 |
|  | 43 | 1908 | 26.0 |  | 1869 | 37.3 | 0.399 |
| **Histology** |  |  |  |  |  |  |  |
| Histology score | 43 | 5.0 | 0.8 |  | 5.0 | 1.0 | 0.976 |
|  |  |  |  |  |  |  |  |
| **Functional tests** |  |  |  |  |  |  |  |
| Cylinder rearing |  |  |  |  |  |  |  |
| RFP | 15 | 0.73 | 0.07 |  | 0.71 | 0.06 | 0.853 |
|  | 36 | 0.66 | 0.04 |  | 0.72 | 0.04 | 0.343 |
| Novel object recognition | |  |  |  |  |  |  |
| D1-1h (s) | 15 | 0.2 | 0.2 |  | 0.3 | 0.3 | 0.812 |
| D1-24h (s) | 15 | -0.2 | 0.2 |  | 0.0 | 0.3 | 0.463 |
| D1-1h (s) | 36 | 0.3 | 0.1 |  | 0.3 | 0.1 | 0.830 |
| D1-24h (s) | 36 | 0.3 | 0.2 |  | 0.1 | 0.1 | 0.254 |
| ET-1h (s) | 15 | 5.2 | 1.5 |  | 5.3 | 2.0 | 0.963 |
| ET-24h (s) | 15 | 3.9 | 0.9 |  | 2.9 | 1.0 | 0.502 |
| ET-1h (s) | 36 | 24.4 | 3.2 |  | 23.3 | 3.4 | 0.815 |
| ET-24h (s) | 36 | 19.2 | 3.4 |  | 13.5 | 2.4 | 0.193 |
