# Supplementary material for: Transient effect of melatonin treatment after neonatal hypoxic-ischemic brain injury in rats
Source: PLoS One. 2019 Dec 20;14(12):e0225788. doi: 10.1371/journal.pone.0225788 (PMC6924669; doi:10.1371/journal.pone.0225788)
Supplement: Attachment — Submitted filename: Response to reviewers.docx [file pone.0225788.s003.docx]

# Response to reviewers

We would like to thank the reviewers for their time spent reviewing the manuscript and for their valuable comments. We have responded to all issues and questions raised the by the editor and reviewer and made necessary changes to the manuscript and figures to accommodate your critique. See specific answers to each point raised below.

## Journal Requirements:

*Please ensure that your manuscript meets PLOS ONE's style requirements, including those for file naming.*

We have gone over the manuscript and made sure it complies with the PLOS style requirements.

*1. We note that you have stated that you will provide repository information for your data at acceptance. Should your manuscript be accepted for publication, we will hold it until you provide the relevant accession numbers or DOIs necessary to access your data. If you wish to make changes to your Data Availability statement, please describe these changes in your cover letter and we will update your Data Availability statement to reflect the information you provide.*

The data has been deposited in the repository and the DOI is provided in the cover letter.

*2. We note that you have included the phrase “data not shown” in your manuscript. Unfortunately, this does not meet our data sharing requirements. PLOS does not permit references to inaccessible data. We require that authors provide all relevant data within the paper, Supporting Information files, or in an acceptable, public repository. Please add a citation to support this phrase or upload the data that corresponds with these findings to a stable repository (such as Figshare or Dryad) and provide and URLs, DOIs, or accession numbers that may be used to access these data. Or, if the data are not a core part of the research being presented in your study, we ask that you remove the phrase that refers to these data.*

We have now included the data in a supplementary table (S2 Table) and included a reference to that in the manuscript.

## Reviewer #1:

*Berger et al. describe the effects of melatonin (MEL) treatment in a model of hypoxia-ischemia (HI) in the P7 rat.
While this is an interesting research topic (considering the safety of the molecule and clinical settings) we feel that the manuscript need several improvements before it can be accepted.

- In the introduction consider more recent works on melatonin in clinical settings: Biran et al. 2019 Int J mol Sci; Colella et al., 2016, Early Hum Dev 102: 1-3; Biran et al., 2014, 56: 717-723.*

Thank you for this comment. References to the suggested work has been included in the introduction section in the manuscript.

*- Again, in the discussion, consider several papers reporting that MEL promotes myelination with oligodendroglial maturation (Olivier et al., PlosOne 2009, 4, e7128) and decreases white matter inflammation (Villapol et al., Ped Res 2011, 69:51-55) in neonatal rats after cerebral injury.*

Thank you for bringing our attention to these papers. A reference to Villapol *et al* has been added to the discussion regarding short term effects. The study by Olivier *et al* provides compelling evidence of mechanisms of melatoninergic neuroprotection. We feel, however, that the choice of animal model (e17 uterine artery ligation) makes the result more relevant to a slightly different clinical problem (diffuse white matter injury in very preterm infants) than we attempt to address (perinatal hypoxia-ischemia).

*- As in your study you also shown that HI induced demyelination, it could has been interesting to measure the thickness of myelin (in the corpus callosum) in the 3 groups of animals, and to evaluate whether MEL was associated to an increase in the density of mature oligodendrocytes.*

Thank you for this suggestion. We have now included the measurements of corpus callosum in all three groups in the results (Figure 4, and results section under white mater injury development in cc) and commented on this in the discussion. The results indicate a slight tendency towards better preservation of the cc among MEL animals, but the difference was not near statistical significance and cc thickness was foremost correlated with the overall brain injury. Unfortunately, we are not able to perform further immunohistochemistry to investigate further the oligodendrocyte density and maturity.

*- WM injury is the underlying cause of motor and cognitive disability in injuried babies that suffer HI and/or ischemia. Evaluation of sensorimotor performance and motor coordination by using the pole test could have been a good test to see (or not) an effect of MEL on HI (namely in mild injured animals).*

Thank you for this suggestion. We chose to evaluate sensorimotor performance using the cylinder rearing test based on extensive use of this test in previous neonatal HI research and ease of comparison to previous work. The test is sensitive to unilateral lesions in all parts of the corticospinal tract (Schallert T, Fleming SM, Leasure JL, Tillerson JL, Bland ST *Neuropharmacology.* 2000;**39**:777–87.). Normalization of test performance follows restoration of corticospinal tract function in the neonatal mouse (van Velthoven CTJ, van de Looij Y, Kavelaars A, Zijlstra J, van Bel F, Huppi PS, et al *Ann Neurol.* 2012;**71**:785–96.). A disadvantage of this test is that it depends on lateralization of sensorimotor function, but since the Vannucci model is (mostly) unilateral, the test is well suited to this model in our opinion. The addition of a less specific test such as the pole test could perhaps increase the chance of discovering non-lateralized sensorimotor dysfunction. We will keep this in mind when designing experiments in the future.

*- Concerning no sex-difference in this study allows to concider MEL as a broad spectrum treatment.*

Thank you for this comment. We agree that this study does not provide evidence against recommending melatonin to either males or females. However, as discussed under “Sex differences in melatoninergic neuroprotection”, we do not feel that our study can answer the question of sex-dependent effects with a high degree of confidence, due to the skewed grouping of males and females in the treatment groups. A sentence addressing this has been added to the discussion.
